# Continuous Long-Term Exposure to Low Concentrations of MWCNTs Induces an Epithelial-Mesenchymal Transition in BEAS-2B Cells

**DOI:** 10.3390/nano11071742

**Published:** 2021-07-01

**Authors:** Hélène Barthel, Christian Darne, Laurent Gaté, Athanase Visvikis, Carole Seidel

**Affiliations:** 1 Institut National de Recherche et de Sécurité, CEDEX, F-54519 Vandœuvre-lès-Nancy, France; helene.barthel@inrs.fr (H.B.); christian.darne@inrs.fr (C.D.); laurent.gate@inrs.fr (L.G.); 2 Ingénierie Moléculaire et Physiopathologie Articulaire (IMoPA), Biopôle, Campus Biologie Santé, UMR 7365 CNRS-Université de Lorraine, CEDEX, F-54000 Vandœuvre-lès-Nancy, France; athanase.visvikis@univ-lorraine.fr

**Keywords:** nanotoxicology, MWCNTs, BEAS-2B cells, epithelial-mesenchymal transition

## Abstract

In the field of nanotechnology, the use of multi-walled carbon nanotubes (MWCNTs) is growing. Pulmonary exposure during their production, use, and handling is raising concerns about their potential adverse health effects. The purpose of this study is to assess how the physical characteristics of MWCNTs, such as diameter and/or length, can play a role in cellular toxicity. Our experimental design is based on the treatment of human bronchial epithelial cells (BEAS-2B) for six weeks with low concentrations (0.125–1 µg/cm^2^) of MWCNTs having opposite characteristics: NM-403 and Mitsui-7. Following treatment with both MWCNTs, we observed an increase in mitotic abnormalities and micronucleus-positive cells. The cytotoxic effect was delayed in cells treated with NM-403 compared to Mitsui-7. After 4–6 weeks of treatment, a clear cellular morphological change from epithelial to fibroblast-like phenotype was noted, together with a change in the cell population composition. BEAS-2B cells underwent a conversion from the epithelial to mesenchymal state as we observed a decrease in the epithelial marker E-cadherin and an increased expression of mesenchymal markers N-cadherin, Vimentin, and Fibronectin. After four weeks of recovery, we showed that the induced epithelial-mesenchymal transition is reversible, and that the degree of reversibility depends on the MWCNT.

## 1. Introduction

Over the past few decades, the use of engineered nanomaterials (ENMs) has moved from research to industrial goods production. Among the existing ENMs, carbon nanotubes (CNTs) are cylinders composed of a single (SWCNT) or multiple (MWCNT) rolled graphene sheets. They have been developed for their innovative technical properties linked to their physicochemical characteristics including size, structure, or specific surface area [1]. These hollow cylinders have mechanical and electrical properties which allow numerous applications in different industrial fields such as electronics, aeronautics and medical applications [2,3,4,5]. These ENMs help to meet new technological challenges and appear to be of major economic interest. However, the increased use of CNTs has raised concerns about the health risks associated with occupational exposure to aerosolized materials emitted during handling and industrial processes. The main exposure route for workers is inhalation by which CNTs can reach the most distal areas of the pulmonary system. Because of their density and size, they can reach deep into the lungs where the airway epithelium is the first line of contact with the inhaled materials [6].

Since CNTs are produced with different lengths and diameters depending on their application, their toxicity may vary considerably, and the potential health risks posed by MWCNTs are not fully known and are still being investigated. For this reason, an increasing number of in vitro and in vivo studies have been devoted to the evaluation of the pulmonary toxicity of MWCNTs. Studies performed in rodents show that MWCNTs exposure may lead to an inflammatory response and, depending on their physical and chemical properties, the development of fibrosis, granulomas, or lung cancer [7,8,9,10,11,12,13,14]. Experiments carried out in cell lines highlight genotoxic effects, transforming potential, and cytotoxicity [7,15,16,17,18]. The physical interaction between CNTs and the mitotic spindles remains a potential cause of cellular toxicity. In fact, abnormal multipolar mitoses trigger the mis-segregation of chromosomes or DNA damage [19,20]. As the correct course of mitosis is a key part of cellular development and the maintenance of genetic material, CNTs could be then responsible for cellular genotoxicity by perturbing the nuclear division process. DNA damages as well as abnormal mitoses are events that can promote the initiation of carcinogenesis. In this multi-step process, it was demonstrated that the epithelial-mesenchymal transition (EMT) takes place in neoplastic cells that have previously undergone genetic or epigenetic changes in oncogenes and tumour suppressor genes [21,22,23]. EMT is known to be a reversible and dynamic cellular program whereby epithelial cells progressively become mesenchymatous [24,25]. This conversion involves deep phenotypic remodelling, accompanied by a switch in gene expression from “epithelial genes” such as E-cadherin (*cdh1*), to “mesenchymal genes”, such as N-cadherin (*cdh2*), Vimentin (*vim*), or Fibronectin (*fn1*) [21]. This transition can occur because of genetic modification, in response to cellular stress or DNA damage, but also as a result of inflammation and the initiation of a fibrotic process. The constitutive expression of EMT inducers can also maintain a mesenchymal phenotype while allowing the metastatic cells to survive by repression of cell death mechanisms [26]. In this way, the EMT would therefore play a role during the early stages of carcinogenesis, but also during later stages during the metastasis process as it confers to cells motility, invasion, and migration ability [22]. It has been shown that MWCNTs induced EMT in human epithelial cells [27,28]. It was also observed that long MWCNTs, which interact with type II alveolar cells, lead to the loss of E-cadherin and the increase of fibronectin [29]. Furthermore, TGF-β1-mediated EMT was triggered by the treatment of human bronchial epithelial cells with MWCNTs [30,31].

While numerous toxicological studies have been performed with acute high concentrations of MWCNTs, the effect of low concentrations of MWCNTs remains less certain. In this study, we have selected two MWCNTs with contrasting length and diameter to evaluate the influence of these physical characteristics on the toxicity: the long and thick Mitsui-7 and the short and thin NM-403. We investigated the morphological and phenotypical changes induced by the two MWCNTs in the human bronchial epithelial cell line, BEAS-2B. To do so, we designed a schedule of treatments with low concentrations of MWCNTs (0.125–1 µg/cm^2^) for six consecutive weeks followed by a recovery period of four weeks.

## 2. Materials and Methods

### 2.1. Carbon Nanotubes and Dispersion Protocol

The Mitsui-7 (MWNT-7/NRCWE-006) from Mitsui (Lot# 05072001K28; Tokyo, Japan) was a kind gift from the National Research Centre for the Working Environment (Copenhagen, Denmark) [32]. The NM-403 was obtained from the Joint Research Centre (Ispra, Italy). The shapes of the CNTs were observed by transmission electron microscopy (TEM, JEOL 2100-F) and are presented in Supplementary Figure S1A. These MWCNTs have been fully characterized previously [33,34] and their main physical and chemical characteristics are presented in Table 1. Only Mitsui-7 has been classified as “possibly carcinogenic to humans” by the International Agency for Research on Cancer (IARC, 2017). Mitsui-7 and NM-403 were dispersed in LHC-9-BSA 1% medium at a final concentration of 0.5 mg/mL, vortexed for 15 s and sonicated 15 min at 40% amplitude using a Branson Sonifier S-450 D equipped with a cup-horn device (Branson Ultrasonics Corp., Danbury, CT, USA). MWCNTs dispersion was appreciated using a light microscope (Supplementary Figure S1B). MWCNTs were freshly prepared before each treatment.

### 2.2. Cell Culture and Treatment

**Figure 1:**
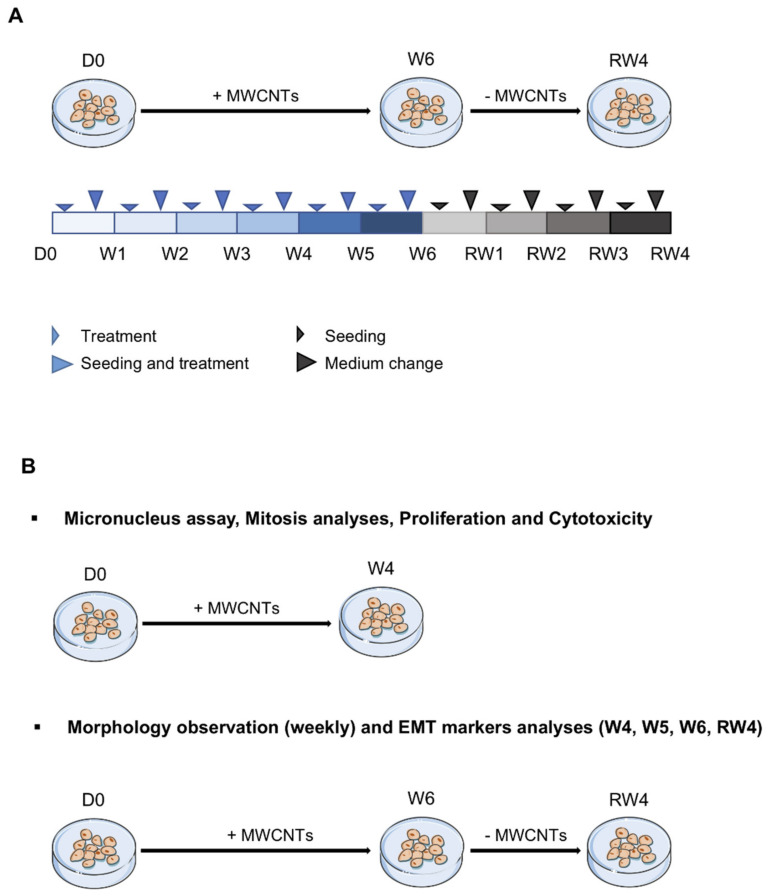
Scheme of the treatment schedule for BEAS-2B cells. (**A**) The cells were seeded at 3500 cells/cm^2^ on day 0. Cells were treated with 0.125, 0.25 and 0.5 μg/cm^2^ of Mitsui-7 or 0.25, 0.5 and 1 μg/cm^2^ of NM-403 twice a week for 6 weeks followed by 4 weeks without treatment. (**B**) Micronucleus assay, abnormal mitoses, cell proliferation and cytotoxicity were evaluated the first four weeks (W1 to W4). Cell morphology was observed weekly from the 1st week of treatment to the 4th week of the recovery period. Analysis of EMT markers using RT-qPCR and Flow Cytometry was conducted after 4 to 6 weeks of treatment (W4 to W6) and after the 4th week of the recovery period (RW4). EMT: epithelial-mesenchymal transition, RW: recovery week, W: week.

The human bronchial epithelial cell line BEAS-2B was obtained from the ATCC (American Type Culture Collection, Manassas, VA, USA, ^®^CRL-9609). The cells were maintained in an LHC-9 medium (Gibco^TM^ #12680013) at 37 °C in a humid atmosphere with 5% CO_2_. The cells were seeded at 3500 cells/cm^2^ and treated either with vehicle (LHC-9-BSA 1%) or MWCNTs to the final concentrations of 0.125, 0.25 and 0.5 µg/cm^2^ of Mitsui-7 or 0.25, 0.5 and 1 µg/cm^2^ of NM-403. Higher concentrations of Mitsui-7 were too cytotoxic to achieve an exposure of 6 consecutive weeks. The conversion to material per culture medium volume (µg/mL) is presented in Supplementary Table S1. The cells were seeded on Day 0, then subcultured and treated for 6 consecutive weeks, followed by 4 weeks without treatment corresponding to the recovery period. Once a week every Monday, the cells were washed with PBS 1× and treated, while once a week every Friday, the cells were washed with PBS 1× and detached with trypsin-EDTA (Gibco^TM^ #25200056). The activity of the trypsin was inhibited by adding 1 volume of DTI (Defined Trypsin Inhibitor, Gibco^TM^ #R007100). The cells were then seeded at the same cellular density and treated. The treatment schedule is described in Figure 1.

### 2.3. Proliferation and Cytotoxicity

Real-time imaging of cultured cells was performed by using the IncuCyte S3^®^ device (Essen BioScience, Royston, UK). Cell proliferation was determined by using the NucLight Rapid Red Reagent (Essen BioScience #4717, reagent that labeled total cells) and the Cytotox Green Reagent (Essen BioScience #4633, reagent that labeled dead cells). The ratio of dead cells/total cells enabled the cytotoxicity to be evaluated. Cell proliferation as well as cytotoxicity were evaluated during the first 4 weeks of treatment.

### 2.4. Abnormal Mitosis and Micronucleus Analysis

Cells were seeded and treated in culture chambers (Sarstedt #94.6150.201), rinsed with PBS 1× and fixed for 15 min with 2% formaldehyde in PBS. The cell membranes were permeabilised for 8 min with 0.1% Triton × 100 in PBS. The cells were then washed with PBS, incubated at room temperature for one-hour intervals with anti-α-Tubulin (Cell Signaling #3873, 1:1000) and then Alexa-Fluor-488 anti-mouse (Invitrogen #A11029, 1:1000) sequentially, while protected from light. Nuclei were stained with DAPI (ProLong^TM^ Gold Antifade Mountant with DAPI, Invitrogen # P36931). The analysis of abnormal mitoses was performed in 200 mitotic cells. Mitoses were classified as “normal” when their mitotic spindle was made up of two poles, whereas they were classified as “monopolar” or “multipolar” when one or more than two poles could be observed, respectively. The micronucleus assay was performed by analysing 2000 cells, during which we counted cells with micronuclei. Treatment of cells for 24 h with 1.25 µg/cm^2^ of vanadium pentoxide (V_2_O_5_, Sigma #223794) was used as a positive control. V_2_O_5_ was suspended in distilled H_2_O and sonicated in an ice-cooled cup-horn device for 15 min prior to its addition to the culture media.

### 2.5. Morphological Observations

Cellular morphology was observed weekly using light microscope at the 10× objective. The morphology was categorized as “normal”, “needle-shaped”, or “mixed” during the 10 weeks of the assay. These observations were summarized to create a heat map, where normal shapes are presented in light blue, mixed in a medium tone of blue and needle-shapes in dark blue.

### 2.6. Analysis of Cell Populations

After detachment by trypsin, the cells (1 × 10^6^) were fixed by adding ice-cold ethanol 70% in droplets under constant agitation. The sample was stored at −20 °C for at least 2 h before analysis. The fixed cells were washed with 1 mM EDTA in PBS and centrifuged at 350× *g* for 5 min. The cell pellet was suspended in PBS 1× and analysed using an AccuriC6 flow cytometer (BD Biosciences, le Pont de Claix, France). Each sample was analysed at the rate of 10,000 events counted in the total cells population gate. FlowJo^TM^ (v10.7.1.) software was used to analyse the SSC:FSC profiles.

### 2.7. Total RNA Isolation and RT-qPCR

The RNAs were extracted from 1 × 10^6^ cells using the NucleoSpin^®^ miRNA kit (Macherey-Nagel #740971) in accordance with the manufacturer’s recommendations. Total RNA (500 ng) was reverse transcribed with the iScript cDNA Synthesis Kit^®^ (Bio-Rad #1708890). The cDNAs were diluted to 1/10th in RNase-free water. Real-time PCR was performed with SsoAdvanced™ Universal SYBR^®^ Green Supermix (Bio-Rad #1725272) and 250 nM of forward and reverse primers specific to the genes of interest, i.e., E-cadherin (*cdh1*), N-cadherin (*cdh2*), Vimentin (*vim*), Fibronectin (*fn1*), and to reference genes, i.e., β–actin (*actb*) and GAPDH (*gapdh*) (Eurogentec, sequences of primers are available on request) in a CFX96 Touch^TM^ (Bio-Rad). Amplification was performed as follows: 5 min at 95 °C, 35 cycles (15 s at 95 °C and 60 s at 60 °C). PCR results were analysed using the 2^−ΔΔ*C*t^ method.

### 2.8. EMT Markers Analysis

The cell pellet (1 × 10^6^ cells) was washed with PBS 1× and suspended in 100 μL of buffer (0.5% BSA, 1 mM EDTA-PBS). The E-cadherin-PE (Miltenyi #130-099-688) and Mouse IgG1-PE (Miltenyi #130-113-762) antibodies as well as viability marker Viobility^TM^ (Miltenyi #130-110-207) were added in accordance with the supplier’s recommendations. The cells were incubated for 10 min at room temperature while protected from light, centrifuged for 10 min at 350× *g* and washed with the buffer. The samples were then analysed with the AccuriC6 flow cytometer. Dead cells were excluded from the analysis. The viability of all samples was greater than 99%. Isotype controls allowed the exclusion of non-specific signals in the channel of interest. Data were analysed using FlowJo^TM^ (v10.7.1.) software. Immuno-labeling of E-cadherin was assessed following the same protocol as for α-Tubulin labeling using anti-E-cadherin (Cell Signaling #3195, 1:200) and Alexa-Fluor-568 anti-rabbit (Invitrogen #A11036, 1:1000) antibodies and DAPI (Invitrogen, Carlsbad, CA, USA) for nuclei staining.

### 2.9. Statistical Analysis

Statistical analysis was performed using R-Studio software. A logarithmic transformation was applied to the data when residuals normality and homoscedasticity conditions were not met. A mixed linear regression model was used to test the “concentration” fixed effect, including a random effect termed “experience”. When the “concentration” effect was significant, a multiple-comparison post-hoc test (Dunnett) was applied to test the difference between the control and different levels of concentration. The statistical significance threshold was set at 5%. All the data are presented as mean ± standard error of the mean (SEM).

## 3. Results

### 3.1. Antiproliferative and Cytotoxic Effect of MWCNTs

**Figure 2:**
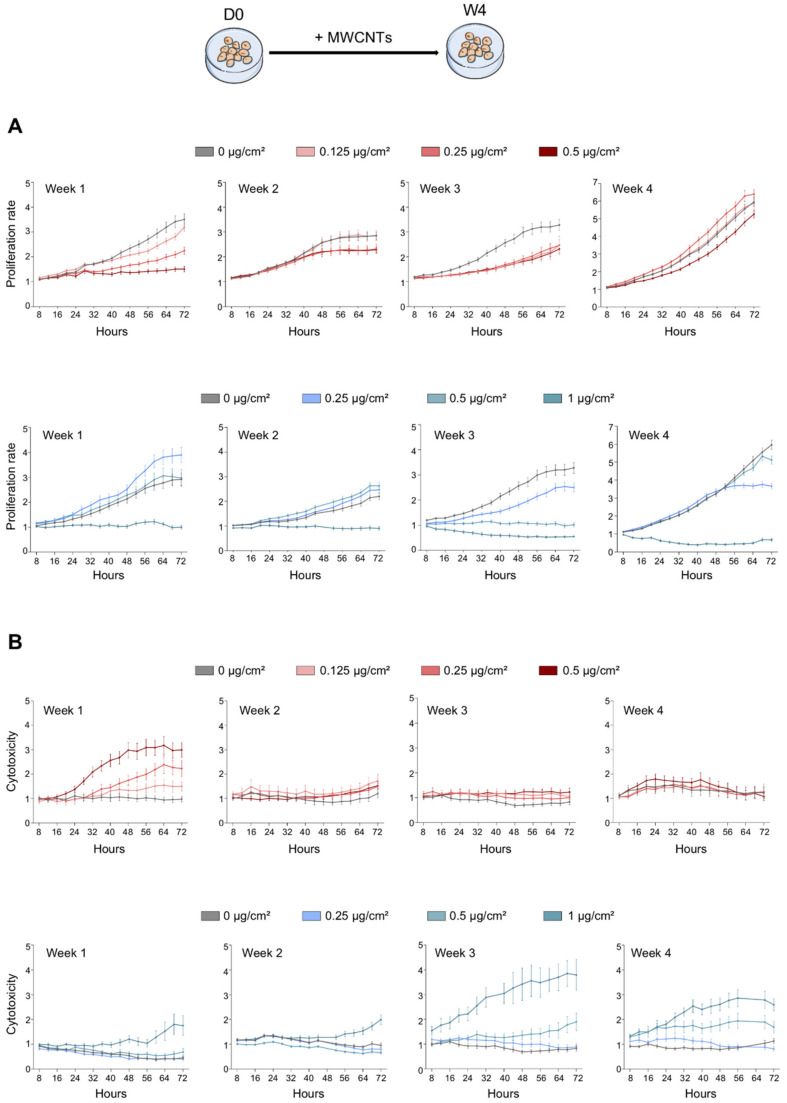
Antiproliferative and cytotoxic effect of MWCNTs. BEAS-2B cells were treated with Mitsui-7 (**upper**) or NM-403 (**lower**) at the indicated concentrations for 4 weeks. (**A**) Proliferation was assessed using a NucLight probe, which labeled total cells. (**B**) Cytotoxicity assay was assessed using Cytotox and NucLight probes and the ratio of dead cells/total cells was determined. Data were normalized to the first point of acquisition after addition of the dyes (4 h). The graphs are representative of three independent experiments.

To assess the impact of MWCNT treatment on cell proliferation and cytotoxicity, the number of total cells as well as dead cells were counted at regular intervals using live-cell imaging. As depicted in Figure 2A (upper panel), treatment of cells with Mitsui-7 decreased the proliferation of cells during the 1st week of treatment, depending on the concentration. The highest concentration of Mitsui-7 also decreased the proliferation rate during the following weeks of treatment, while in the 4th week, the proliferation of cells treated with the intermediate concentration showed the same profiles as the control cells. Mitsui-7 induced a cytotoxic effect during the 1st week of treatment, depending on the concentration, while no cytotoxicity was evident during the following weeks (Figure 2B, upper panel). In NM-403 treated cells, the two lowest concentrations (0.25 and 0.5 µg/cm^2^) did not impact cell proliferation during the first two weeks of treatment (Figure 2A, lower panel). An antiproliferative effect was first observed for the concentration of 0.5 µg/cm^2^ after 3 weeks of treatment. The highest concentration (1 µg/cm^2^) induced a strong decrease in cellular proliferation, which began in the 1st week and continued until the 4th week of treatment. This concentration induced a moderate cytotoxic effect from the 1st week and persisted throughout the following weeks of treatment (Figure 2B, lower panel). However, lower concentrations did not induce cell death during the first two weeks. It is only from the 3rd week that a cytotoxic effect first appeared, depending on the concentration, and persisted until the 4th week.

### 3.2. Abnormal Mitoses and Genotoxicity

**Figure 3:**
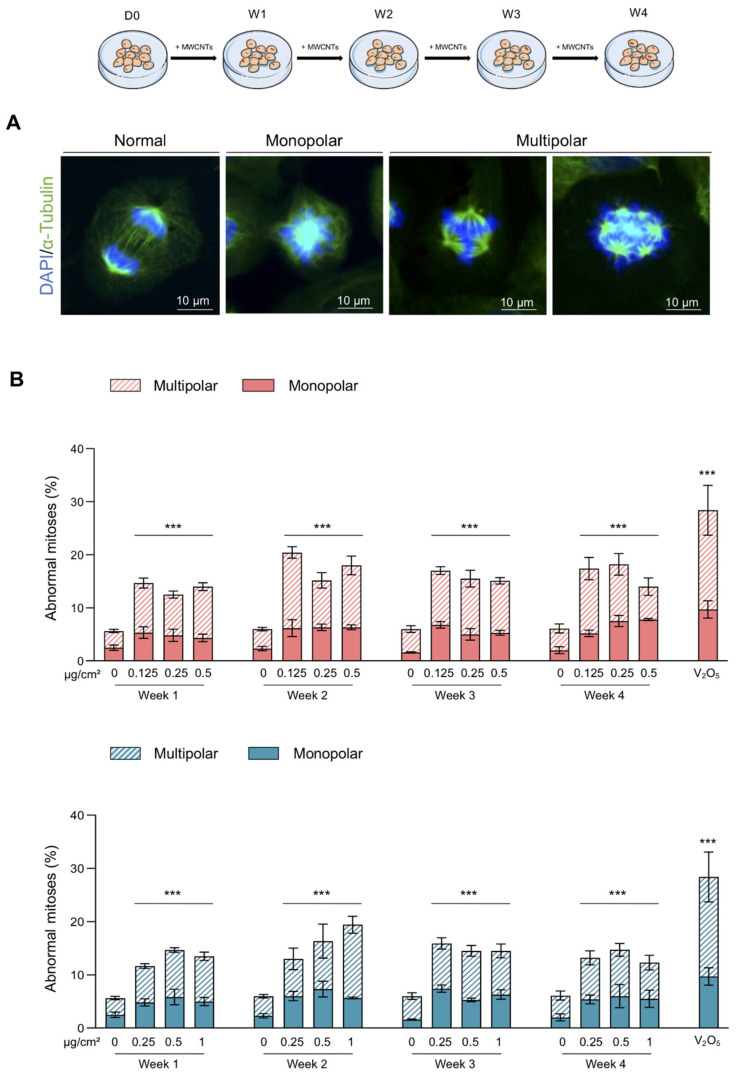
Abnormal mitoses induced by treatment with Mitsui-7 and NM-403. (**A**) The pictures represent a normal mitosis and abnormal mitoses with a monopolar or multipolar spindle. (**B**) The cells were treated with Mitsui-7 (**upper panel**) or NM-403 (**lower panel**) at the indicated concentrations for 4 weeks. The cells were immunolabeled with an anti-α-Tubulin antibody and the nuclei were stained with DAPI. Mitotic spindles were analysed within 200 dividing cells per sample. The histograms indicate the percentage of abnormal mitoses: dashed bars for multipolar and solid bars for monopolar mitoses. A treatment with vanadium pentoxide (V_2_O_5_, 1.25 µg/cm^2^) for 24 h was used as a positive control. The histograms represent the mean ± standard error of the mean (SEM) of three independent experiments. *** *p* < 0.001 significantly different from the control.

**Figure 4:**
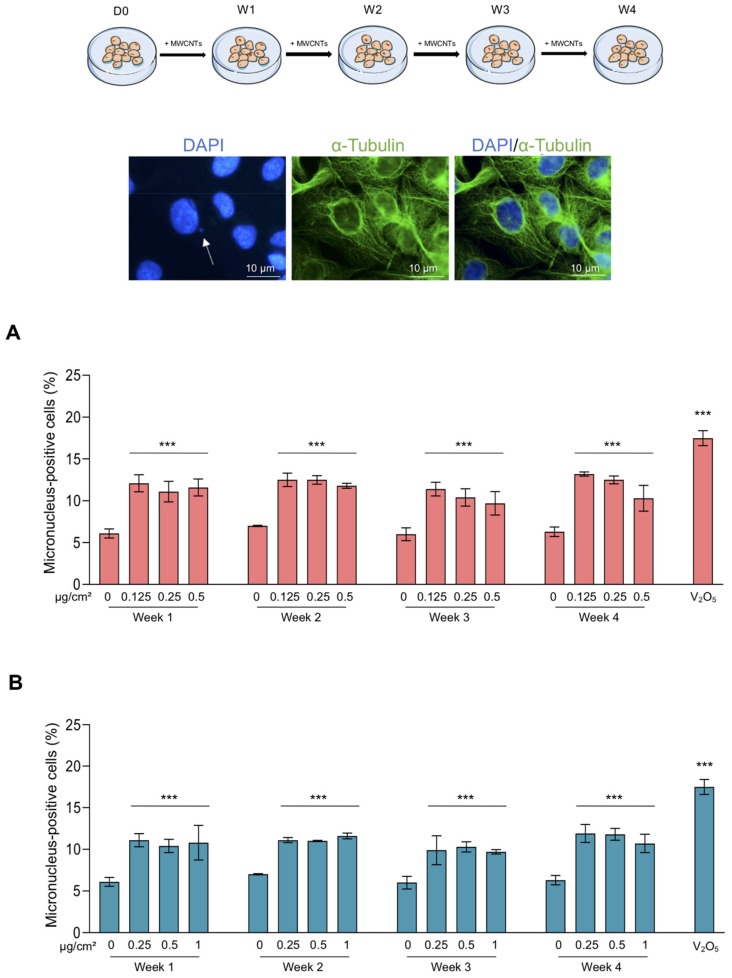
Treatment with Mitsui-7 and NM-403 increased the occurrence of micronuclei. The cells were treated with (**A**) Mitsui-7 (0.125, 0.25 and 0.5 μg/cm^2^) or (**B**) NM-403 (0.25, 0.5 and 1 μg/cm^2^) for 4 weeks. The cells were immunolabeled with an anti-α-Tubulin antibody and the nuclei were stained with DAPI and 2000 cells were analysed in each sample. The fluorescent microscopy images represent a micronucleus following DAPI staining (white arrow). The histograms indicate the percentage of micronucleus-positive cells. A treatment with vanadium oxide (V_2_O_5_, 1.25 µg/cm^2^) for 24 h was used as a positive control. The histograms represent the mean ± standard error of the mean (SEM) of three independent experiments. *** *p* < 0.001 significantly different from the control.

Since treatments with MWCNTs have an antiproliferative effect and the cell division is an important part in the maintenance of genetic integrity, we assessed their impact on mitoses. The different stages of mitosis of BEAS-2B cells were observed (Supplementary Figure S2A) and allow us to detect the presence of CNTs close to dividing cells (Supplementary Figure S2B). Figure 3A represents an example of “normal”, “monopolar” and “multipolar” mitotic cells. Results presented in Figure 3B demonstrate that both MWCNTs increased the number of abnormal mitoses, 2–4 times depending on the treatment, when compared to control cells. The increase in abnormal mitoses was independent of the duration of treatment and the concentration. Indeed, the lowest concentration of MWCNTs increased the percentage of abnormal mitoses by approximately the same extent as the highest concentration and no difference was found during the four weeks of observation. We noticed that the abnormal mitoses were mainly multipolar. V_2_O_5_ was used as positive control and it induced a six-fold increase in abnormal mitoses. Alteration of the mitotic spindle plays a major role in causing genomic instability by promoting the mis-segregation of chromosomes that can be the cause of micronucleus formation. We then evaluated the genotoxic effect of MWCNTs on BEAS-2B cells through the micronucleus test. Both Mitsui-7 and NM-403 significantly increased (two-fold) the frequency of micronucleus-positive cells even at the lowest concentrations tested and from the 1st week of treatment (Figure 4A,B). Similarly, the micronucleus rate induced by MWCNTs was independent of the concentration and the duration of treatment. V_2_O_5_ was used as a positive control. A 24 h-treatment with this agent resulted in a four-fold increase in micronucleus-positive cells compared to the vehicle. Beyond four weeks of treatment, the cells underwent a change in morphology and grew in a disorganized manner, which prevented the analysis of mitoses and the micronucleus assay.

### 3.3. MWCNTs Altered the Cellular Morphology of BEAS-2B Cells

**Figure 5:**
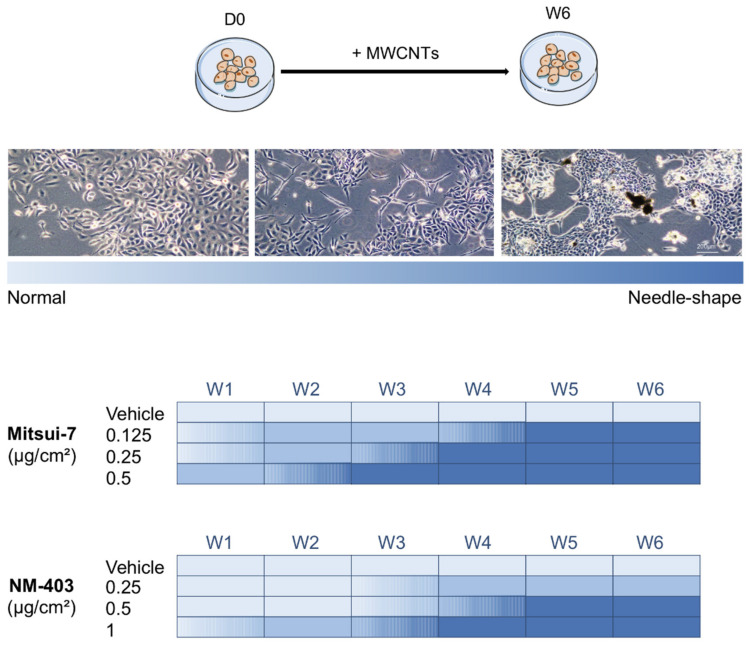
Morphological changes of BEAS-2B cells induced by Mitsui-7 and NM-403. The cells were treated with Mitsui-7 (0.125, 0.25 and 0.5 μg/cm^2^) or NM-403 (0.25, 0.5 and 1 μg/cm^2^) for 6 weeks. Cell morphology was observed weekly with an optical microscope at the 10× objective. Representative pictures of morphological changes are presented from normal to needle-shaped cells (upper panel). The heatmap represents the morphological changes, light blue for cells with a normal shape to dark blue for cells with a needle shape (lower panel). The results are representative of at least three independent experiments. W: weeks.

**Figure 6:**
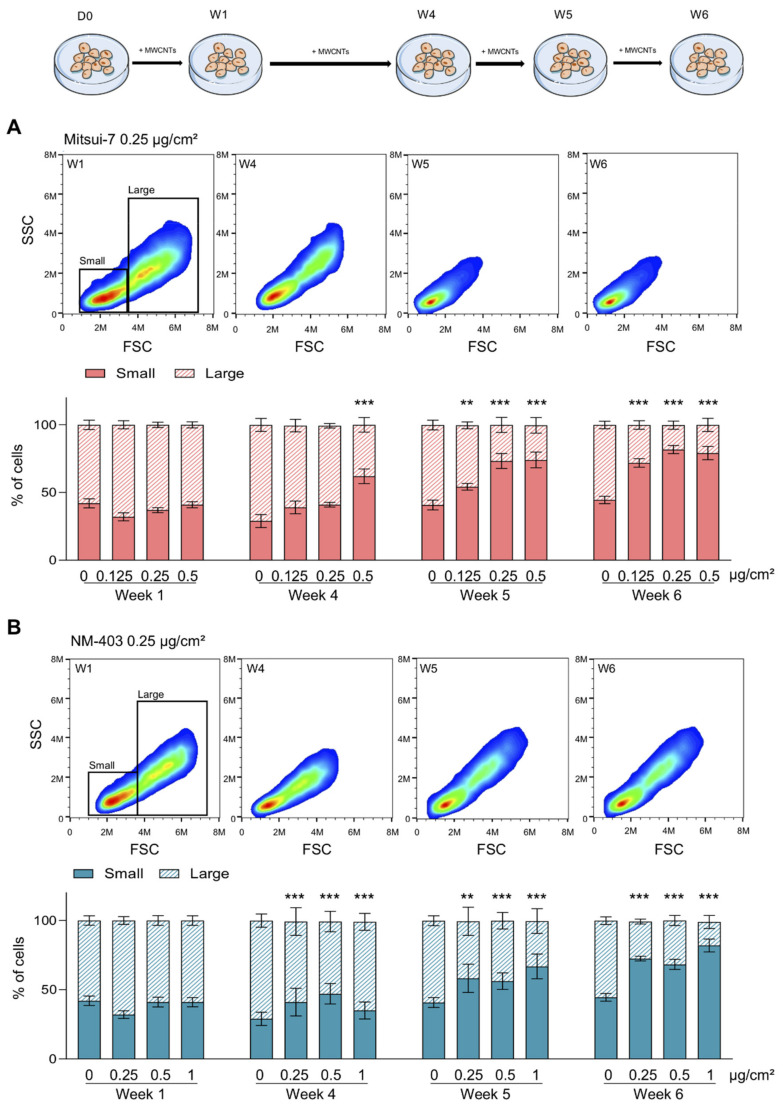
Mitsui-7 and NM-403 induced the selection of a cell population. The cells were treated with (**A**) Mitsui-7 (0.125, 0.25 and 0.5 μg/cm^2^) or (**B**) NM-403 (0.25, 0.5 and 1 μg/cm^2^) and their size and granulometry were analysed using flow cytometry. Based on the SSC:FSC profiles, two cell populations were determined: those of a small size and low granulometry (small) and those of a large size and high granulometry (large). The SSC:FSC profiles from W1, W4, W5 and W6 are represented for Mitsui-7 0.25 µg/cm^2^ and NM-403 0.25 µg/cm^2^. The histograms represent the mean ± standard error of the mean (SEM) of the quantification of the two cell populations for three independent experiments. ** *p* < 0.01, *** *p* < 0.001 significantly different from the control.

BEAS-2B are large adherent cells with a diameter around 20 µm, which form a monolayer under standard culture conditions. We noticed that the cells exposed to both MWCNTs progressively lost their epithelial morphology in favour of smaller elongated and needle-shaped cells that grew in multilayers (Figure 5, upper panel). Mitsui-7-treated cells started to become disorganised depending on the concentration and the duration of treatment. While large changes in cell morphology were observed during the 1st week of treatment with the highest concentration, the changes in cell shape became evident much later (3rd to 4th week) with the two lowest concentrations (Figure 5, middle panel). Treatment with NM-403 also induced the same morphological changes that were clearly evident during the 2nd week (1 µg/cm^2^) or the 4th week (0.5 µg/cm^2^, Figure 5, lower panel). At the lowest concentration of NM-403 used, much fewer cells were observed with altered morphology even after 6 weeks of treatment. The weekly observations of BEAS-2B cells are detailed in Supplementary Figure S3. Because the majority of the treated cells changed their morphology from the 4th week of treatment, we focused on characterizing this population during weeks 4–6. Flow cytometry analyses demonstrated two distinct cell populations: one of a large size and high granulometry, called large cells, and a second of a small size and low granulometry, called small cells (Figure 6). The quantification of the number of cells composing each population indicates that control cells are composed of about 40% of small and 60% of large cells (Figure 6). In accordance with the morphological changes, treatment of cells with both MWCNTs inverted this ratio. Indeed, we observed an increase in small cells following treatment with Mitsui-7, depending on concentration and duration of treatment, an effect that began during the 3rd week of treatment (Supplementary Figure S4A) and which reached 80% with the highest concentration during the 6th week (Figure 6A). Similarly, treatment with NM-403 led to an increase in small cells that began during the 3rd week (Supplementary Figure S4B) while the cell population was composed of between 70% and 80% small cells by the 6th week, independent of the concentration (Figure 6B).

### 3.4. MWCNTs Are Responsible for the Epithelial-Mesenchymal Transition

**Figure 7:**
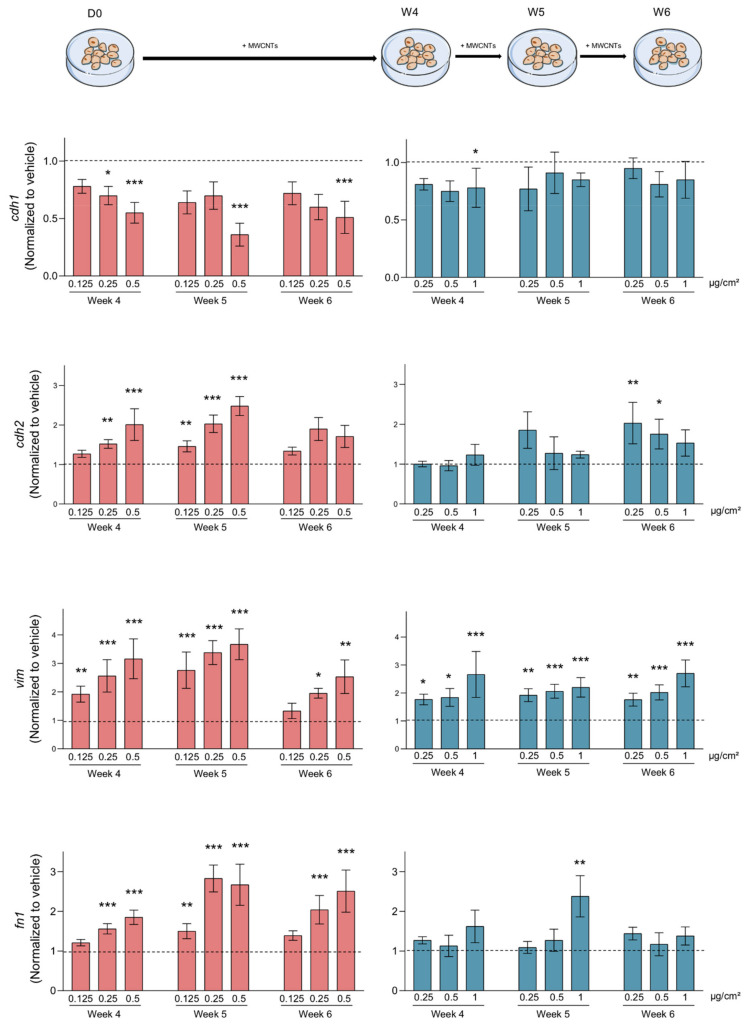
MWCNTs-induced EMT marker expression. RNAs from total cells treated with the indicated concentration of Mitsui-7 (**left**) or NM-403 (**right**) were extracted and gene expression was analysed using RT-qPCR. The epithelial marker E-cadherin (*cdh1*) and mesenchymal markers N-cadherin (*cdh2*), Vimentin (*vim*) and Fibronectin (*fn1*) were evaluated after 4, 5 and 6 weeks of treatment. The histograms represent the mean ± standard error of the mean (SEM) for three independent experiments. * *p* < 0.05, ** *p* < 0.01, *** *p* < 0.001 significantly different from the control.

**Figure 8:**
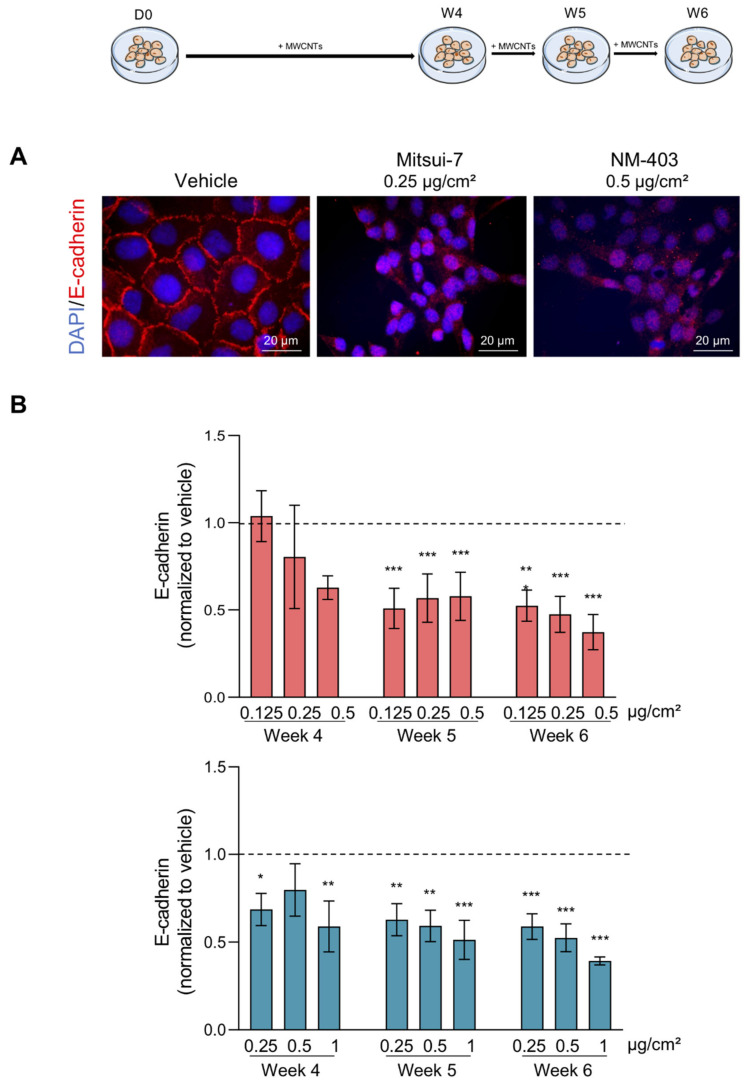
MWCNTs reduced E-cadherin in BEAS-2B cells. (**A**) The cells were treated with vehicle, 0.25 µg/cm^2^ of Mitsui-7 or 0.5 µg/cm^2^ of NM-403 for 6 weeks. The cells were immunolabeled with an anti-E-cadherin antibody and the nuclei were stained with DAPI. (**B**) Cells treated with the indicated concentrations of Mitsui-7 or NM-403 for 4 to 6 weeks were labeled with the anti-E-cadherin antibody and analysed using flow cytometry. The extracellular marker E-cadherin was quantified in the population of small cells and the bar graph represents the ratio of mean fluorescence intensity/total number of cells reported in the vehicle. The histograms represent the mean ± standard error of the mean (SEM) for three independent experiments. * *p* < 0.05, ** *p* < 0.01, *** *p* < 0.001 significantly different from the control.

The phenotypic modifications we have described are similar to those observed in mesenchymal cells and suggest that BEAS-2B cells undergo a progressive conversion from the epithelial to mesenchymal state. Therefore, we studied the change in the expression of markers during this transition. We observed a decrease of the expression of the epithelial marker E-cadherin (*cdh1*, two-fold) in Mitsui-7 treated cells but not following NM-403 treatment (Figure 7). Consistent with the implementation of an EMT, MWCNT treatments were responsible for the increased expression of mesenchymal-related genes: N-cadherin (*cdh2*, 1.1 to 2.5-fold), Vimentin (*vim*, 1.9 to 3.5-fold) and Fibronectin (*fn1*, 1.5 to three-fold, Figure 7). This increase in the expression of the three genes was more evident from the 4th week of treatment with Mitsui-7 at the highest concentration (Figure 7). Treatment with NM-403 resulted in a moderate induction, mainly observed in the 6th week. Next, we analysed E-cadherin expression at the protein level. Western blot analyses of this protein showed a decreased expression six weeks after treatment with Mitsui-7 and NM-403 (Supplementary Figure S5). In accordance with the mRNA level, treatment with CNTs also induced Vimentin at the protein level (Supplementary Figure S5). In a qualitative approach using immunofluorescence, we observed a decrease in the E-cadherin level in both Mitsui-7 and NM-403 treated cells (Figure 8A). Next, we quantified E-cadherin protein in the emerging population of small cells using flow cytometry. After four weeks of treatment with Mitsui-7, no change in E-cadherin expression was observed in these cells (Figure 8B). After five and six weeks of treatment, its protein level was strongly diminished (between 30% and 60%), depending on the concentration of Mitsui-7 used. Treatment with NM-403 also decreased E-cadherin expression by 50%, which was mainly observed during the 5th and 6th weeks (Figure 8B). Taken together, these results provide evidence for the implementation of an EMT in BEAS-2B cells after treatment with Mitsui-7 and NM-403.

### 3.5. Reversibility of the Effects

**Figure 9:**
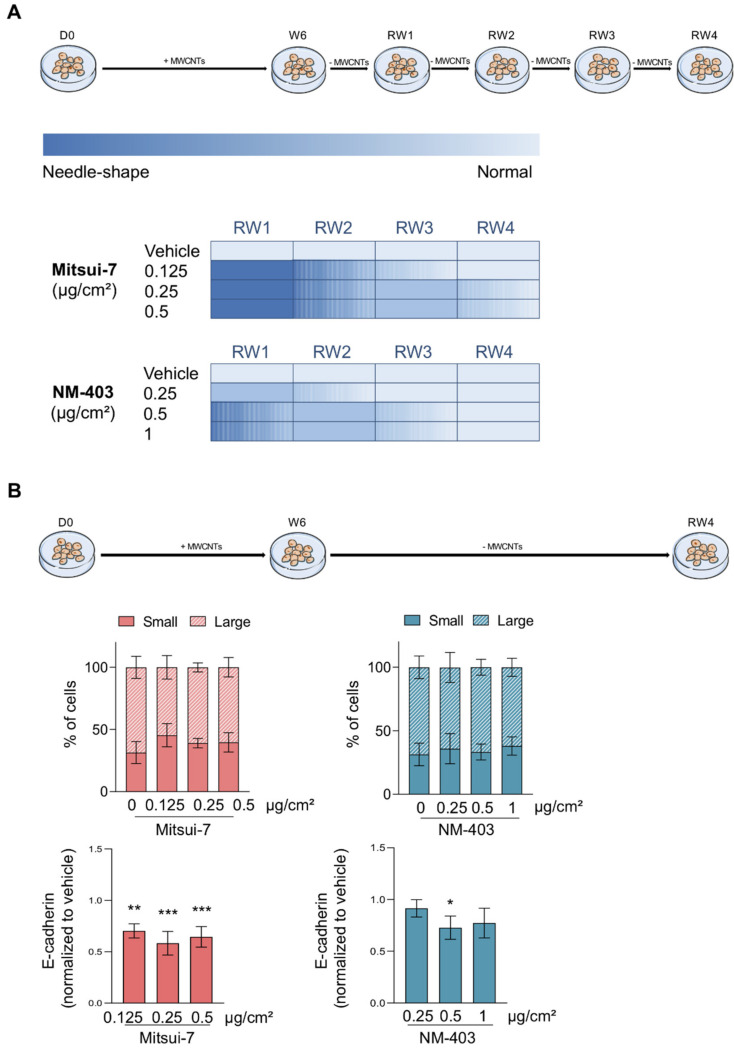
The MWCNTs-induced EMT is reversible. After the 6 weeks of treatment with Mitsui-7 (0.125, 0.25 and 0.5 μg/cm^2^) or NM-403 (0.25, 0.5 and 1 μg/cm^2^), the cells were cultured without treatment for 4 weeks (RW1-RW4). (**A**) The heatmap represents the morphological changes observed in cells during the 4 weeks of the recovery period from needle shape (dark blue) to normal shape (light blue). (**B**) The two cellular populations (small and large cells) were analysed using flow cytometry and determined based on SSC:FSC profiles during the RW4. The extracellular marker E-cadherin was quantified in the population of small cells using flow cytometry and the bar graph represents the ratio of mean fluorescence intensity/total number of cells reported to the vehicle. The histograms represent the mean ± standard error of the mean (SEM) for three independent experiments. * *p* < 0.05, ** *p* < 0.01, *** *p* < 0.001 significantly different from the control.

In order to assess the reversibility of the effects, the cells treated for six weeks were maintained in culture for four additional weeks without any treatment. During this recovery period, cells progressively changed their morphology from needle- to normal-shape (Figure 9A). In cells treated with the lowest concentration of Mistui-7, this change was observed from the 3rd week of the recovery period, while it took place later for cells treated with the higher concentrations (Figure 9A). The cells treated with NM-403 returned to a normal morphology more rapidly. The weekly observations of BEAS-2B cells are detailed in Supplementary Figure S6. For cells treated with NM-403 at the lowest concentration, the change to normal-shape was observed from the 2nd week of the recovery period and from the 3rd week for cells treated with the highest concentration. These results are in accordance with the composition of the cell population quantified by flow cytometry, 40% of small cells, 60% of large cells after four weeks of recovery, a level similar to those of untreated cells (Figure 9B). At the end of the recovery period, the E-cadherin expression in the small cell population remained lower than in control cells, although it tends to increase when compare to cells after six weeks of treatment (Figure 9B). As expected, we observed at the end of the recovery period, that E-cadherin protein in the total cell population increased and returned close to the level in control cells, while a decrease of the Vimentin protein level was noticed (Supplementary Figure S7).

## 4. Discussion

In this in vitro study, we evaluated and compared the toxic potential, at low concentrations, of two MWCNTs with opposite physical characteristics during a 6-week exposure. Human epithelial cells were continuously exposed to MWCNTs, thus simulating the effects of a chronic exposure. We first demonstrated that both MWCNTs were responsible for abnormal mitoses. It is well established that the structure of the mitotic spindle is essential for the proper distribution of genetic material between two daughter cells [35]. In the current experiments, treatment with Mitsui-7 and NM-403 increased the occurrence of abnormal mitoses 2–4 times from the 1st up to the 4th week of treatment. It has been shown that the long and thick Mitsui-7 can interact with the actin and tubulin filaments, leading to cytokinesis blockage [7,36,37]. Other studies have demonstrated that SWCNTs and MWCNTs are causing abnormal mitoses [38,39]. Futhermore, it was demonstrated that MWCNTs were located in the cytoplasm and the nucleus, and had a strong association with the centrosomes [39]. These alterations may be responsible for DNA damages [35]. When we treated BEAS-2B cells with MWCNTs, a significant increase in micronucleus formation was recorded. Thus, the two MWCNTs have a similar genotoxic effect, suggesting that their size does not influence their genotoxic potential in vitro. Those results correspond to the increase of micronucleus following a 24-h treatment of human cells with long MWCNTs [40,41]. However, we did not observe concentration or time effects, which suggests that this effect had reached a plateau at the lowest concentration. One could argue that if the hypothesis that CNTs behave as tubulin analog is true, as soon as they enter into the nucleus the CNTs induce cellular abnormalities. These effects could be seen starting at the first concentration of CNTs if the numbers of internalized NMs are sufficient enough. Then, no concentration effect would be seen. In addition, the cellular penetration kinetics is important and the effect observed after the first week of treatment may not require additional treatment except for selection pressure.

We found micronucleus in treated cells and this genotoxic effect induced by MWCNTs can lead to cellular toxicity. In the proliferation and cytotoxicity profiles, we noticed that the cells treated with Mitsui-7 suffered from cytotoxic disturbance during the 1st week of treatment, whereas cells treated with NM-403 suffered from the same cytotoxic disturbance during the 3rd and 4th weeks of treatment, indicating a delay in the effect between the two MWCNTs. According to these observations and based on the data from the literature, we hypothetize that CNTs interact with mitotic spindle conducing to abnormal mitoses and consequently in micronucleus formation [42]. A part of the altered cells die, early in the schedule, and the other part of cells could escape to cell cycle control points, modulate their transcriptome leading to a progressive modification of their phenotype. Indeed, we observed that BEAS-2B cells gradually lost their epithelial morphology (large cuboid cells growing in monolayer) in favour of the so-called “needle-shaped” population (small cells with elongated cytoplasm and nucleus). The cellular plasticity observed during the treatments reflects the capacity of the cells to adapt to a stressful environment. Interestingly, such morphological changes have been also reported before, Chae et al. have shown a “spindle-like” and fibroblastic morphologic change in BEAS-2B cells and a loss of epithelial cell markers and a gain of mesenchymal cell markers in the case of non-small cell lung cancer (NSCLC) research [43]. Treatment with MWCNTs, accompanied with morphological changes, decreased E-cadherin at the mRNA and protein level, which is a hallmark of EMT [21,23]. Globally, the expression of mesenchymal markers N-cadherin, Vimentin and Fibronectin was increased, which supports the implementation of this process [22,24]. These results are in line with those from in vitro experiments that demonstrated that MWCNTs are responsible for inducing EMT in A549 and BEAS-2B cell lines [27,30]. It has been shown that MWCNT-induced EMT contributes to adverse outcomes such as pulmonary fibrosis, and metastasis in carcinogenesis depending on their length [30]. In addition, long MWCNTs were found to interact directly with type II alveolar epithelial cells (RLE-6TN) in rats, resulting in its loss of E-cadherin and the increase of fibronectin expression thereby inducing EMT [29]. Regarding the selection of the population as well as the establishment of EMT, our results confirm that long and thick MWCNTs induced EMT. This transition took place sooner, with a higher magnitude and at a lower concentration than after treatment with the short and thin CNT NM-403.

Even though both MWCNTs led to an EMT of BEAS-2B cells, the specific surface area is an important physical parameter of nanoparticles, which could influence their toxicity. If we take only into account this characteristic, which is five times lower for Mitsui-7 compared to NM-403, one may have expected that NM-403 would have been more cytotoxic than Mitsui-7. Then, other physical or chemical parameters may be involved in the toxicological properties of these CNTs. Indeed, CNTs length and diameter may play a role in their toxicity. Long and thick CNTs have been shown to be more inflammogenic and carcinogenic than shorter ones [44,45,46]. In addition, because of their expected higher stiffness, long and thick CNTs may alter structural protein organization more efficiently than short and thin CNTs. Cellular uptake kinetics and mechanisms may differ between the two types of these NMs.

To the best of our knowledge, no recovery study has been carried out on BEAS-2B cells following treatment with MWCNTs. Our results showed that during the recovery period the cells gradually regain their epithelial morphology and probably follow a mesenchymal-to-epithelial transition. However, after four weeks of recovery, the vast majority of the cell population appears to have returned to a normal epithelial phenotype, the remaining small cells still show a decrease in E-cadherin protein. Our results are in good agreement with the study of Li et al. that showed an EMT-memory of NSCLC cells even after the restoration of the epithelial phenotype [47]. In our model, it would be interesting to determine whether cells previously treated with MWCNTs may have EMT-memory, be more susceptible to other external stresses, and could degenerate more quickly and irreversibly.

## 5. Conclusions

MWCNTs are responsible for inducing both abnormal mitoses and micronucleus formation, regardless of their length and diameter. Morphological changes and EMT were established with both MWCNTs. However, these deleterious effects come up earlier and at a lower concentration with the long and thick Mitsui-7 than those observed after the short and thin NM-403 exposure. The reversibility of the transition is also dependent of the length and/or diameter, since it starts earlier with NM-403 than with Mitsui-7. Although their effects are less important than those observed after a long-MWCNT treatment, it is important to note that shorter and thinner CNTs have toxic effects as well. Additional experiments with a larger panel of CNTs with different lengths and diameters are required to better understand the role of these parameters on the onset of EMT in epithelial cells.

## Figures and Tables

**Table 1:** Mitsui-7 and NM-403 characterization ^1^.

	Product Code	Length (µm)	Diameter (nm)	S_BET_ * (m^2^/g)	Purity (%)	Metal Content (wt%)
Mitsui-7	MWNT-7	5.7 (±0.49)	74 (29–173)	26	>99	P_2_O_5_ 0.14, Fe_2_O_3_ 0.08, SO_3_ 0.08, CaO 0.03, MgO 0.01, SiO 0.006, ZnO 0.001, CuO 0.0003
NM-403	JRCNM4003a	0.4 (±0.03)	12 (5–37)	135	>90	Al_2_O_3_ 0.24, MgO 0.19, MnO 0.16, P_2_O_5_ 0.14, CoO 0.12, CaO 0.03, CuO 0.003, Fe_2_O_3_ 0.002, NiO 0.0018, ZnO 0.001

* SBET: Specific surface area determined by Brunauer, Emmett and Teller method, ^1^ [34].

## Data Availability

The data presented in this study are available on request from the corresponding author.

## Supplementary Materials

The following are available online at https://www.mdpi.com/article/10.3390/nano11071742/s1. Table S1: Concentrations of MWCNTs for BEAS-2B treatments, Figure S1: Images of MWCNTs and suspensions, Figure S2: Normal and abnormal mitoses of BEAS-2B cells, Figure S3: Mitsui-7 and NM-403 treatment induced changes in BEAS-2B cell morphology, Figure S4: Cell population after 2 and 3 weeks of treatment with Mitsui-7 and NM-403, Figure S5: E-cadherin and Vimentin protein level after 6 weeks of treatment with Mitsui-7 and NM-403, Figure S6: Mitsui-7 and NM-403 treatment induced changes in BEAS-2B cell morphology, Figure S7: E-cadherin and Vimentin protein level after 4 weeks of the recovery period.
