# Supplementary material for: Continuous Long-Term Exposure to Low Concentrations of MWCNTs Induces an Epithelial-Mesenchymal Transition in BEAS-2B Cells
Source: Nanomaterials (Basel). 2021 Jul 1;11(7):1742. doi: 10.3390/nano11071742 (PMC8308165; doi:10.3390/nano11071742)
Supplement: Supplementary file 1 [file nanomaterials-11-01742-s001.zip › nanomaterials-1262795-supplementary.pdf]

nanomaterials

Article

Continuous Long-Term Exposure to Low Concentrations of MWCNTs Induces an Epithelial-Mesenchymal Transition in BEAS-2B Cells

Supplementary data

Hélène Barthel [1,2], Christian Darne [1], Laurent Gaté [1], Athanase Visvikis [2] and Carole Seidel [1,*]

[1] Institut National de Recherche et de Sécurité, CEDEX F-54519 Vandœuvre-lès-Nancy, France; helene.barthel@inrs.fr (H.B.); christian.darne@inrs.fr (C.D.); laurent.gate@inrs.fr (L.G.)
[2] Ingénierie Moléculaire et Physiopathologie Articulaire (IMoPA), Biopôle, Campus Biologie Santé, UMR 7365 CNRS-Université de Lorraine, Vandœuvre-lès-Nancy, France; athanase.visvikis@univ-lorraine.fr
* Correspondence: carole.seidel@inrs.fr

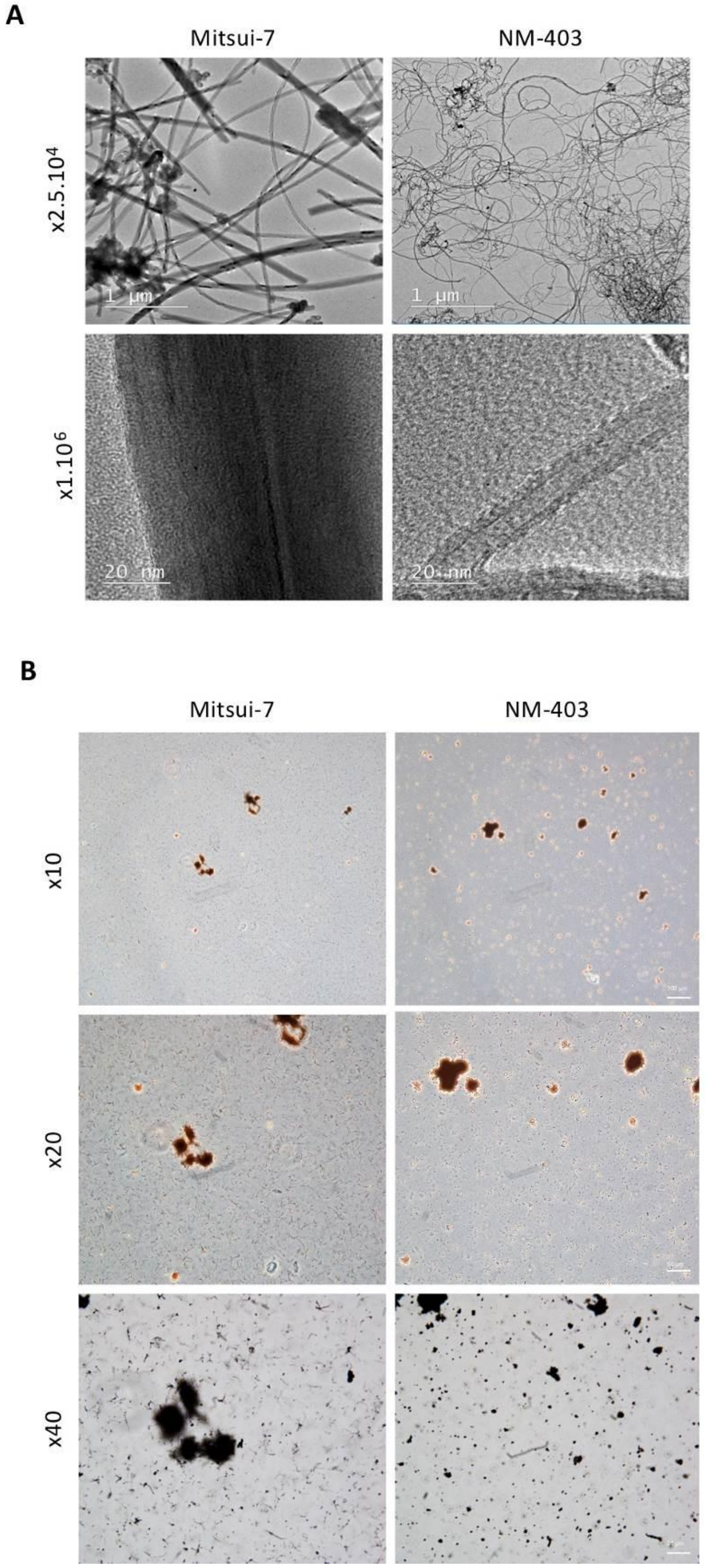

Supplementary figure 1: Images of MWCNTs and suspensions. (A) Transmission electronic microscopy images of Mitsui-7 and NM-403 powder. (B) Phase-contrast images of MWCNT suspensions in BSA 1%-LHC-9 medium after 15 min of sonication.

Supplementary table 1: Concentrations of MWCNTs for BEAS-2B treatments. The cells were cultured in a T75 flask in a final volume of 15 mL.

Concentration (μg/cm^2)	Concentration (μg/mL)
0.125	0.625
0.25	1.25
0.5	2.5
1	5

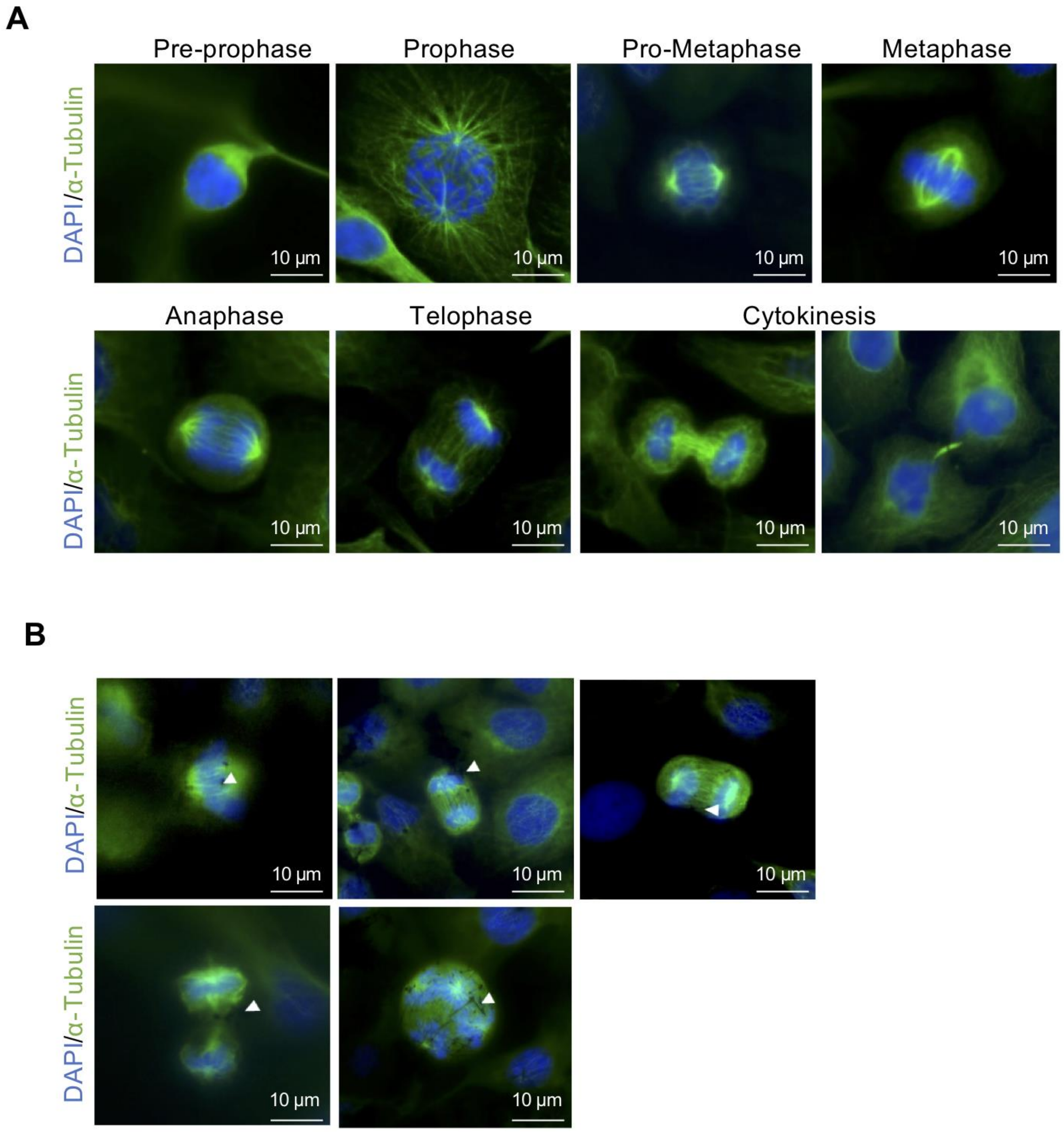

Supplementary figure 2: Normal and abnormal mitoses of BEAS-2B cells. The cells were immunolabelled with α-tubulin-FITC and nuclei stained with DAPI. (A) Images of cells in the different mitosis phases. (B) Following cell treatment by Mitsui-7 or NM-403, carbon nanotubes are visible close to the mitotic spindle and/or nucleus during cell division.

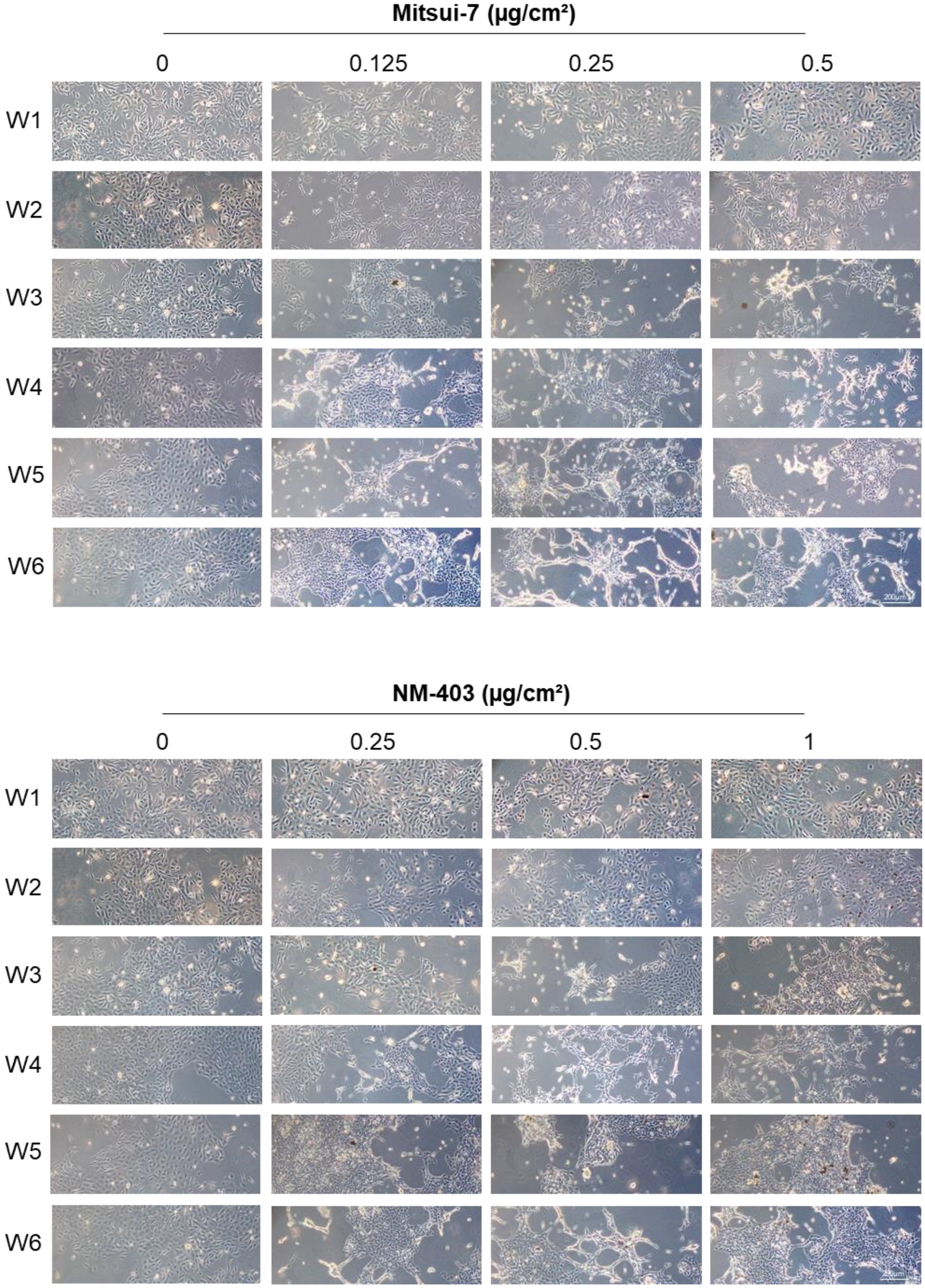

Supplementary figure 3: Mitsui-7 and NM-403 treatment induced changes in BEAS-2B cell morphology. After treatment by the indicated concentration of Mitsui-7 and NM-403, phase-contrast microscopy images were recorded weekly from the 1st (W1) to the 6th (W6) week.

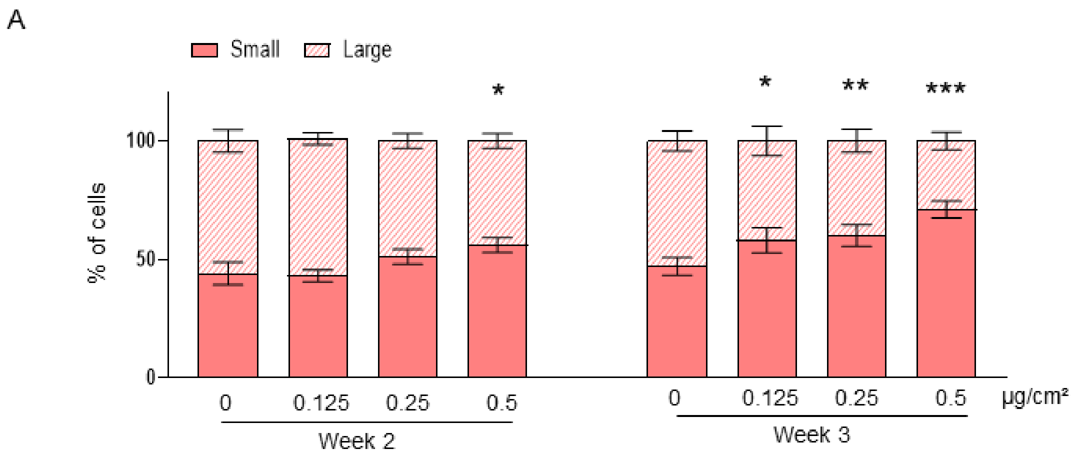

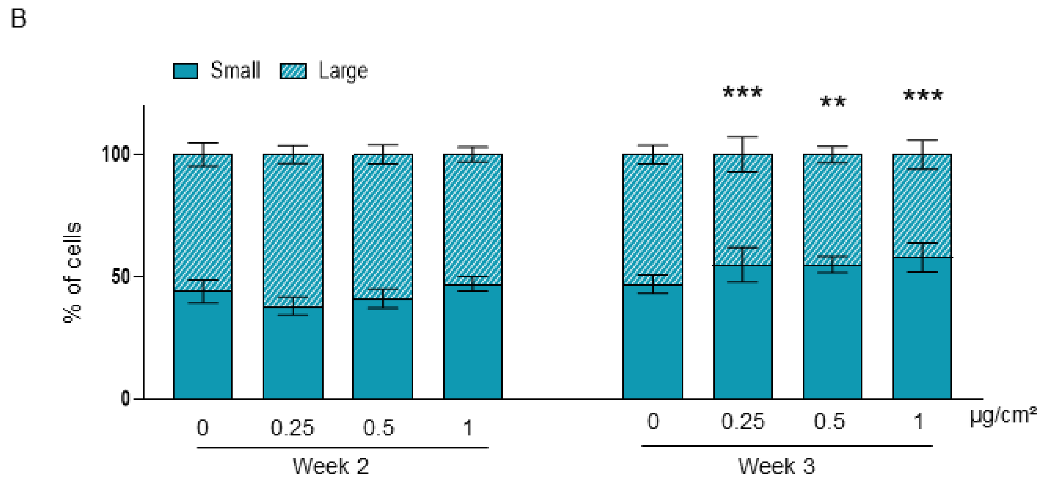

Supplementary figure 4: Cell population after 2 and 3 weeks of treatment with Mitsui-7 and NM-403. The cells were treated with vehicle, (A) Mitsui-7 (0.125, 0.25 and 0.5 μg/cm²) or (B) NM-403 (0.25, 0.5 and 1 μg/cm²) for 2 and 3 weeks and their size and granulometry were analyzed by flow cytometry. Based on SSC:FSC profiles, 2 cell populations were discriminated: with small size and low granulometry (small) or with large size and high granulometry (large). The histograms represents the mean +/- standard error of the mean (SEM) of the quantification of the two cell populations of three independent experiments. * $p<0.05$, ** $p<0.01$, *** $p<0.001$ significantly different from the control.

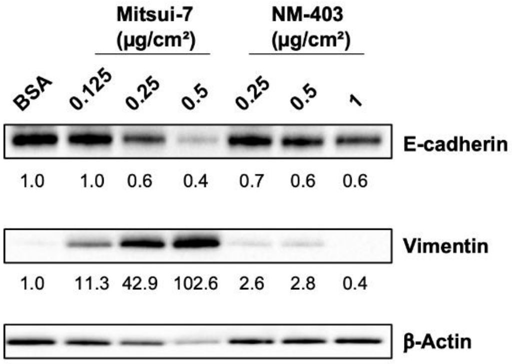

Supplementary figure 5: E-cadherin and Vimentin protein level after 6 weeks of treatment with Mitsui-7 and NM-403. Total proteins were extracted from cells treated 6 weeks at the indicated concentrations of Mitsui-7 and NM-403. E-cadherin and Vimentin protein level were analyzed by Western Blot. Quantifications are presented under protein of interest (Protein of interest/Loading control) reported to the vehicle control. Blots are representative of two independent experiments.

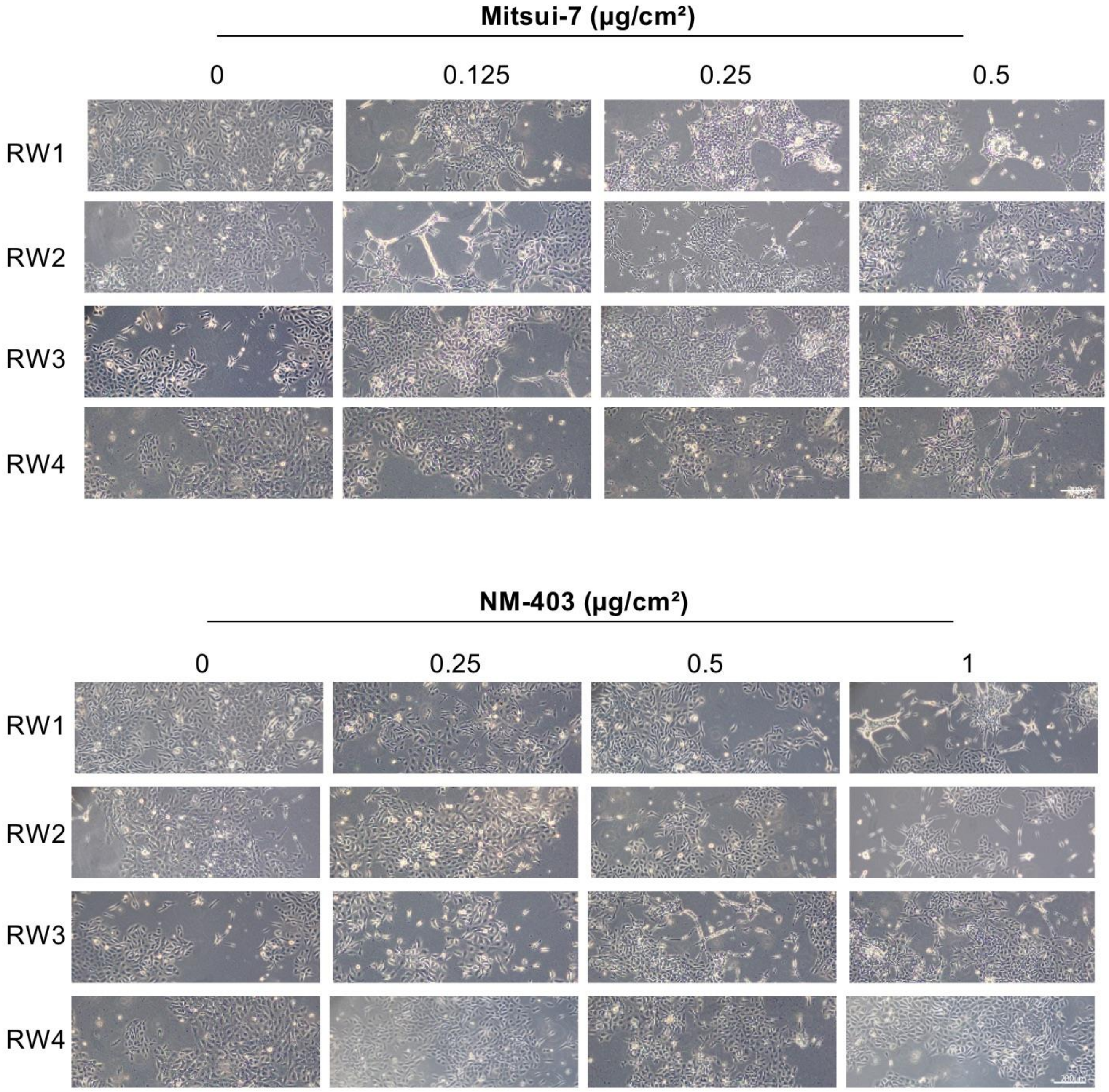

Supplementary figure 6: Mitsui-7 and NM-403 treatment induced changes in BEAS-2B cell morphology. Phase-contrast microscopy images were recorded weekly in the recovery period, from the 1st to 4th week (RW1 to RW4).

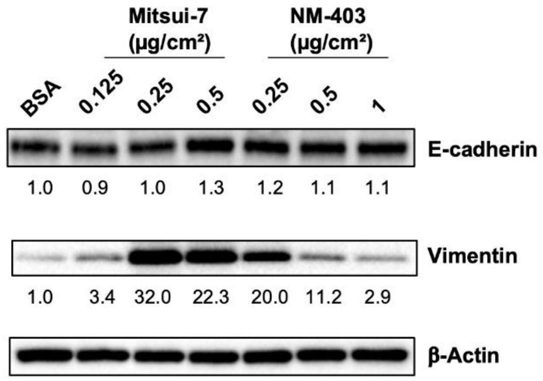

Supplementary figure 7: E-cadherin and Vimentin protein level after 4 weeks of the recovery period. Total proteins were extracted from cells 4 weeks after the recovery period. E-cadherin and Vimentin protein level were analyzed by Western Blot. Quantifications are presented under protein of interest (Protein of interest/Loading control) reported to the vehicle control. Blots are representative of at least two independent experiments.
